# Clinical manifestations and outcome in *Staphylococcus aureus *endocarditis among injection drug users and nonaddicts: a prospective study of 74 patients

**DOI:** 10.1186/1471-2334-6-137

**Published:** 2006-09-11

**Authors:** Eeva Ruotsalainen, Kari Sammalkorpi, Janne Laine, Kaisa Huotari, Seppo Sarna, Ville Valtonen, Asko Järvinen

**Affiliations:** 1 Division of Infectious Diseases, Department of Medicine, Helsinki University Central Hospital, Helsinki, Finland; 2 Department of Medicine, Tampere University Hospital, Tampere, Finland; 3 Department of Public Health, University of Helsinki, Helsinki, Finland

## Abstract

**Background:**

Endocarditis is a common complication in *Staphylococcus aureus *bacteremia (SAB). We compared risk factors, clinical manifestations, and outcome in a large, prospective cohort of patients with *S. aureus *endocarditis in injection drug users (IDUs) and in nonaddicts.

**Methods:**

Four hundred and thirty consecutive adult patients with SAB were prospectively followed up for 3 months. Definite or possible endocarditis by modified Duke criteria was found in 74 patients: 20 patients were IDUs and 54 nonaddicts.

**Results:**

Endocarditis was more common in SAB among drug abusers (46%) than in nonaddicts (14%) (odds ratio [OR], 5.12; 95% confidence interval [CI], 2.65–9.91; *P *< 0.001). IDUs were significantly younger (27 ± 15 vs 65 ± 15 years, *P *< 0.001), had less ultimately or rapidly fatal underlying diseases (0% vs 37%, *P *< 0.001) or predisposing heart diseases (20% vs 50%, *P *= 0.03), and their SAB was more often community-acquired (95% vs 39%, *P *< 0.001). Right-sided endocarditis was observed in 60% of IDUs whereas 93% of nonaddicts had left-sided involvement (*P *< 0.001). An extracardiac deep infection was found in 85% of IDUs and in 89% of nonaddicts (*P *= 0.70). Arterial thromboembolic events and severe sepsis were also equally common in both groups. There was no difference in mortality between the groups at 7 days, but at 3 months it was lower among IDUs (10%) compared with nonaddicts (39%) (OR, 5.73; 95% CI, 1.20–27.25; *P *= 0.02).

**Conclusion:**

*S. aureus *endocarditis in IDUs was associated with as high complication rates including extracardiac deep infections, thromboembolic events, or severe sepsis as in nonaddicts. Injection drug abuse in accordance with younger age and lack of underlying diseases were associated with lower mortality, but after adjusting by age and underlying diseases injection drug abuse was not significantly associated with mortality.

## Background

Bacteremic infections caused by *Staphylococcus aureus *have been reported with increasing frequency, and it has emerged as a leading cause of infective endocarditis (IE) in many regions of the world [1-3]. Endocarditis has been observed in 11% to 35% of *S. aureus *bacteremias (SAB) attributable to different sets of diagnostic criteria [4-6]. *S. aureus *endocarditis is associated with higher occurrence of extracardiac deep infections due to metastatic spread, thromboembolic complications, and a high mortality compared with IE caused by other pathogens [1,2].

The incidence of endocarditis has not been changed during the past two decades [7,8] but classic risk factors such as rheumatic heart disease are being replaced by new groups, including injection drug users (IDUs), elderly patients with degenerative valve disease, patients with intravascular catheter or prosthetic valve, and nosocomial acquisition [2,3,8]. One of the reasons for increased incidence of *S. aureus *in IE is injection drug abuse [9-11]. Colonization *with S. aureus*, HIV-related immunosuppression, female sex, increasing injection drug use frequency, and a history of previous IE are associated with a higher risk for endocarditis [11,12].

Right-sided involvement, younger age, and lack of pre-existing heart disease or other underlying diseases have been thought to explain the better prognosis of *S. aureus *endocarditis among IDUs than in general population [2,13-15]. Therefore, surgery is less frequently needed and shorter antibiotic courses have been used in IDUs [16,17]. Drug abusers tend to have fewer arterial embolic events or strokes than nonaddicts [13,18]. These differences between IDUs and general population have been derived from non-comparative trials, and there are only few studies where the clinical picture of *S. aureus *endocarditis in these patient groups has been compared [2,13,14,19].

We collected prospectively a large patient population with SAB and compared the risk factors, clinical, and echocardiographic findings as well as outcome of endocarditis in IDUs and in general population.

## Methods

### Patient population

Prospectively randomized adult patients with blood culture positive for *S. aureus *from 5 university hospitals and 7 tertiary care hospitals in Finland were taken into the trial from January 1999 to May 1999 and from January 2000 to August 2002. Originally, the trial was designed for evaluation on the effect of fluoroquinolone (trovafloxacin or levofloxacin) given in addition to most effective standard treatment in SAB [20] [see Additional file 1]. Clinical, pathological, and echocardiographic data of patients with definite or possible IE according to modified Duke criteria were collected [21], and these cases were included into the analysis. The protocol was approved by the ethics committees of all study sites, and written informed consent was obtained from all patients or their representatives. The trial was conducted in accordance with the Declaration of Helsinki.

The exclusion criteria were age younger than 18 years, imprisonment, proven or suspected pregnancy, breastfeeding, epilepsy, another bacteremia during the previous 28 days, polymicrobial bacteremia (≥ 3 microbes), history of allergy to any quinolone antibiotic, previous tendinitis during fluoroquinolone therapy, prior fluoroquinolone use for more than 5 days before randomization, positive culture for *S. aureus *only from a central intravenous catheter, meningitis, neutropenia (< 0.5 × 10^9^/L), or failure to supply an informed consent. Patients with bacteremia due to methicillin-resistant *S. aureus *(MRSA) and a *S. aureus *strain resistant to any fluoroquinolone were also excluded.

### Definitions

IDUs were defined as patients who had injected drugs within the past 6 months before randomization. Routine bacteriological methods were used to detect *S. aureus *growth in blood [20] [see Additional file 1]. Bacteremia was hospital-acquired if the first positive blood culture was obtained ≥ 48 hours after admission, or the patient was a resident in a long-term care facility or attended hemodialysis within the preceding 2 months. Prognosis or severity of underlying diseases were classified as healthy, nonfatal, ultimately, or rapidly fatal according to the criteria of McCabe and Jackson [22]. Severe sepsis at the time of first blood culture positive for *S. aureus *was defined as an infectious process leading to organ dysfunction or signs of hypoperfusion or hypotension [23].

The infection focus was documented by clinical, bacteriological, radiological, or pathological investigations. Intravenous catheter-associated bacteremia was defined using the guidelines of the Infectious Diseases Society of America [24]. Extracardiac deep infection was defined as pneumonia, deep-seated abscess, osteomyelitis, septic arthritis, meningitis, septic thrombophlebitis, mediastinitis, urinary tract infection, infection of any extracardiac prosthetic device, or recurrent SAB. Clinical findings of IE such as documentation of a new valvular regurgitant murmur, or evidence of any vascular phenomena such as infarction and embolic event or intracranial hemorrhage were detected. Endocarditis of prosthetic valve was classified as early when occurring ≤ 60 days after valve replacement and as late > 60 days after valve replacement. Leucocytosis was determined if white blood cell count was over 12 × 10^9^/L and leucopenia less than 4 × 10^9^/L.

### Echocardiography

Two-dimensional imaging from multiple tomographic planes and spectral Doppler and color flow imaging were used in all study sites. Transthoracic echocardiography (TTE) and/or transesophageal echocardiography (TEE) as clinically indicated were performed at the discretion of the treating physician and by experienced echocardiographers. The presence of cardiac vegetations, oscillation, paravalvular or intracardiac abscess, new valvular regurgitation, prosthetic valve dehiscence, and valve perforation were recorded [21].

### Antibiotic treatment

The detailed information of the antimicrobial agents, doses, indications and duration of treatment have been described in our former article [20] [see Additional file 1]. All patients with endocarditis were assigned to receive initially cloxacillin or dicloxacillin (2 g q4 h) for 4 to 6 weeks intravenously. Alternatively, cefuroxime (1.5 g q6 h) or vancomycin (1 g bid) were allowed if a contraindication against the use of semisynthetic penicillins was noted. Aminoglycoside (either tobramycin or netilmicin at 1 mg/kg of body weight q8h) was added to the drug therapy described above for the first 7 days. Rifampicin (450 mg once daily for patients under 50 kg and 600 mg once daily for patients over 50 kg in weight, orally or intravenously) was recommended for at least 4 weeks when an endocarditis or an extracardiac deep infection was suspected or confirmed. Half of the patients were randomized to receive a fluoroquinolone either trovafloxacin or levofloxacin in addition to therapy above. Fluoroquinolone treatment was not observed to have an impact on mortality in SAB or in patients with IE [20] [see Additional file 1]. In cases of renal dysfunction the antibiotic doses were adjusted as recommended by the manufacturers.

### Clinical outcome

All patients were prospectively followed for 3 months after the first positive blood culture for *S. aureus*. Case fatality rates were recorded at 7 days and 28 days, and at 3 months. Other outcome measures were site of valvular involvement, evidence of extracardiac deep infections or thromboembolic events, duration of fever (recorded in days until axillary temperature was < 37.5 C°), duration of hospitalization and need for cardiac surgery. Laboratory tests were conducted on the day of the first positive blood culture for *S. aureus*.

### Statistical analysis

Statistical analyses were performed with SPSS^® ^version 12.0. (SPSS Inc., Chicago, IL, USA). Univariate analyses for categorical variables were calculated with chi-square test or Fisher's exact test as appropriate, and for continuous variables the Mann-Whitney test was used. Odds ratios (OR) with 95% confidence intervals (CI) were as association measures. Cox regression analysis was used to compare survival of IDUs with nonaddicts, and results were given as hazard ratios (HR) with 95% CI. Survival was calculated from the day of the first blood culture positive for *S. aureus *until 3 months. All tests were 2-tailed and *P *values of < 0.05 were considered significant.

## Results

### Patient characteristics

During the study period, 430 patients with *S. aureus *bacteremia were included into this trial. The included patients represented 31% of all SAB patients in study hospitals. Endocarditis was observed in 74 of 430 patients (17%), of whom 20 were IDUs and 54 were nonaddicts. Thus, IE was observed in 46% of all 44 IDUs and in 14% of all 386 nonaddicts with SAB (OR, 5.12; 95% CI, 2.65–9.91; *P *< 0.001). Patients with endocarditis differed from those with SAB only by having significantly more often a predisposing heart disease or a pre-existing liver disease (chronic hepatitis C infection in 19 patients and hepatic cirrhosis in 5 patients), but less preceding trauma (Table 1). Other predisposing characteristics and underlying diseases of the patients with IE did not differ from those with SAB.

**Table 1:** Characteristics of 430 patients with *Staphylococcus aureus *bacteremia with and without endocarditis.

	Bacteremia with endocarditis	Bacteremia without endocarditis	Total			
Characteristic	(*n *= 74)	(*n *= 356)	(*n *= 430)	OR	95% CI	*P*
Age, mean years ± SD	55 ± 22	59 ± 17	58 ± 18	-	-	0.26
Male sex	47 (64)	221 (62)	268 (62)	1.06	0.63–1.79	0.82
Hospital-acquired	34 (46)	198 (56)	232 (54)	0.68	0.41–1.12	0.13
Previous skin disease or wound	34 (46)	189 (53)	223 (52)	0.75	0.45–1.24	0.26
Previous surgery^a^	18 (24)	77 (22)	95 (22)	1.17	0.65–2.10	0.61
Trauma in prior 2 months	12 (16)	98 (28)	110 (26)	0.51	0.26–0.99	0.04
Central intravenous catheter	10 (14)	48 (14)	58 (14)	1.00	0.48–2.09	0.99
Hemodialysis	6 (8)	38 (11)	44 (10)	0.74	0.30–1.82	0.51
Previous *S. aureus *deep infection	3 (4)	28 (8)	31 (7)	0.50	0.15–1.67	0.33
Previous *S. aureus *bacteremia	2 (3)	15 (4)	17 (4)	0.63	0.14–2.82	0.75
Predisposing heart condition	47 (64)	63 (18)	110 (26)	8.10	4.69–13.98	<0.001
Degenerative heart disease	22 (30)	32 (9)	54 (13)	4.28	2.31–7.94	<0.001
Injection drug use	20 (27)	24 (7)	44 (10)	5.12	2.65–9.91	<0.001
Prosthetic valve	17 (23)	1 (0.3)	18 (4)	105.88	13.82–811.08	<0.001
Prior endocarditis	11 (15)	0 (0)	11 (3)	-	-	<0.001
Congenital heart disease	5 (7)	5 (1)	10 (2)	5.09	1.43–18.05	0.02
Mitral valve prolapse	2 (3)	2 (1)	4 (1)	4.92	0.68–35.48	0.14
Rheumatic heart disease	2 (3)	1 (0.3)	3 (1)	9.86	0.88–110.21	0.08
Coronary artery disease	21 (28)	89 (25)	110 (26)	1.19	0.68–2.08	0.54
Corticosteroid use ≥ 1 month	8 (11)	37 (10)	45 (11)	1.05	0.47–2.35	0.92
Immunosuppressive therapy^b^	3 (4)	26 (7)	29 (7)	0.54	0.16–1.82	0.45
Diabetes	19 (26)	89 (25)	108 (25)	1.04	0.58–1.84	0.90
Alcoholism	4 (5)	44 (12)	48 (11)	0.41	0.14–1.17	0.10
Liver disease	24 (32)	43 (12)	67 (16)	3.49	1.95–6.25	<0.001
Malignancy	9 (12)	54 (15)	63 (15)	0.77	0.36–1.65	0.51
Chronic renal failure	8 (11)	52 (15)	60 (14)	0.71	0.32–1.56	0.39
HIV positive	3 (4)	5 (1)	8 (2)	2.97	0.69–12.70	0.14

**NOTE. **Data are no. (%) of patients, unless otherwise indicated.

^a ^Includes cardiac and non-cardiac surgery during 3 months preceding the positive blood culture.

^b ^During 6 months preceding the positive blood culture.

Among patients with endocarditis, IDUs as a group were younger and had less predisposing heart conditions, coronary artery disease or diabetes than nonaddicts (Table 2). When the underlying diseases were grouped by the predicted prognoses (McCabe's classification), none among the IDUs had a rapidly fatal or ultimately fatal disease but they were found in 20 of 54 nonaddicts (37%) (*P *= 0.001). However, there were three IDUs with HIV infection.

**Table 2:** Characteristics of injection drug users and nonaddicts with *Staphylococcus aureus *endocarditis.

	Injection drug users	Nonaddicts	
Characteristic	(*n *= 20)	(*n *= 54)	*P*
Age, mean years ± SD	27 ± 15	65 ± 15	<0.001
Male sex	15 (75)	32 (59)	0.28
Hospital-acquired	1 (5)	33 (61)	<0.001
Previous skin disease or wound	12 (60)	22 (41)	0.19
Previous surgery^a^	0 (0)	18 (33)	0.002
Trauma in prior 2 months	3 (15)	9 (17)	1.00
Central intravenous catheter	0 (0)	10 (19)	0.05
Hemodialysis	0 (0)	6 (11)	0.18
Previous *S. aureus *deep infection	2 (10)	1 (2)	0.18
Previous *S. aureus *bacteremia	2 (10)	0 (0)	0.07
Predisposing heart condition	4 (20)	27 (50)	0.03
Degenerative heart disease	0 (0)	22 (41)	<0.001
Prosthetic valve	0 (0)	17 (32)	0.004
Prior endocarditis	1 (5)	10 (19)	0.27
Congenital heart disease	3 (15)	2 (4)	0.12
Mitral valve prolapse	0 (0)	2 (4)	1.00
Rheumatic heart disease	0 (0)	2 (4)	1.00
Coronary artery disease	0 (0)	21 (39)	<0.001
Corticosteroid use ≥ 1 month	0 (0)	8 (15)	0.10
Immunosuppressive therapy^b^	0 (0)	3 (6)	0.56
Diabetes	1 (5)	18 (33)	0.02
Alcoholism	0 (0)	4 (7)	0.57
Liver disease	18 (90)	6 (11)	<0.001
Chronic hepatitis C	17 (85)	2 (4)	<0.001
Hepatic cirrhosis	1 (5)	4 (7)	1.00
Malignancy	0 (0)	9 (17)	0.10
Chronic renal failure	0 (0)	8 (15)	0.10
HIV positive	3 (15)	0 (0)	0.02

**NOTE. **Data are no. (%) of patients, unless otherwise indicated.

^a ^Includes cardiac and non-cardiac surgery during 3 months preceding the positive blood culture.

^b ^During 6 months preceding the positive blood culture.

Only one drug abuser had a hospital-acquired bacteremia and none associated with the use of the central intravenous catheter (Table 2). Severe sepsis was observed in 9 of 20 IDUs (45%) and in 28 of 54 nonaddicts (52%) without significant difference between the groups (*P *= 0.79).

### Site of endocarditis

According to the modified Duke criteria for endocarditis, 56 patients (76%) were confirmed as definite (10 by pathologic criteria and 46 by clinical criteria), and 18 patients (24%) as possible (Table 3). Left-sided involvement was observed in 93% of nonaddicts whereas in 60% of IDUs had right-sided endocarditis (tricuspid valve involvement in all patients). Among nonaddicts aortic valve was slightly more often involved (44%) than mitral valve (35%).

**Table 3:** Classification, valvular involvement and echocardiographic findings of injection drug users and nonaddicts with *Staphylococcus aureus *endocarditis.

	Injection drug users	Nonaddicts	Total			
Variable	(*n *= 20)	(*n *= 54)	(*n *= 74)	OR	95% CI	*P*
Criteria, classification^a^						
Possible	1 (5)	17 (32)	18 (24)	0.12	0.01–0.93	0.03
Definite	19 (95)	37 (69)	56 (76)	8.73	1.08–70.67	0.03
Valvular involvement^b^						
Left-sided	6 (30)	50 (93)	56 (76)	0.03	0.01–0.14	<0.001
Aortic	2 (10)	24 (44)	26 (35)	0.14	0.03–0.66	0.006
Mitral	3 (15)	19 (35)	22 (30)	0.33	0.08–1.25	0.15
Aortic and mitral	1 (5)	7 (13)	8 (11)	0.35	0.04–3.07	0.44
Right-sided	12 (60)	4 (7)	16 (22)	18.75	4.83–72.73	<0.001
Both sides	2 (10)	0 (0)	2 (3)	-	-	0.07
Echocardiography performed	20 (100)	53 (98)	73 (99)	-	-	1.00
TTE	20 (100)	52 (96)	72 (97)	-	-	1.00
TEE	9 (45)	40 (74)	49 (66)	0.29	0.10–0.84	0.03
Echocardiography^c^						
Vegetation	14 (70)	25 (47)	39 (53)	0.26	0.87–7.84	0.16
Regurgitation^d^	17 (85)	35 (66)	52 (71)	2.91	0.75–11.27	0.15
Valve perforation	1 (5)	0 (0)	1 (1)	-	-	0.27
Paravalvular or intracardiac abscess	1 (5)	2 (4)	3 (4)	1.34	0.12–15.67	1.00
Duration of bacteremia before diagnosis, median days (range)	2 (0–8)	3 (0–28)	3 (0–28)	-	-	0.67

**NOTE. **Data are no. (%) of patients, unless otherwise indicated. TTE, transthoracic echogardiography; TEE, transesophageal echogardiography.

^a ^Classified by modified Duke criteria.

^b ^Includes abnormal echocardiographic manifestations both in native valve or in prosthetic valve.

^c ^Represents initial echocardiographic findings with TTE alone or with both TTE and TEE.

^d ^Regurgitation observed on echocardiogram in any cardiac valve at the time of diagnosis of endocarditis.

Prosthetic valve endocarditis, involving left side only, occurred in 17 of 74 patients (23%) and they all presented in nonaddicts. The most of these patients had an early onset of prosthetic valve IE (12 patients).

### Echocardiographic findings

Echocardiography was performed in 263 of 430 patients (61%) with SAB. Addicts underwent echocardiography significantly more often (91%) compared with nonaddicts (58%) (OR, 7.31; 95% CI, 2.56–20.84; *P *< 0.001). In endocarditis, TTE was performed in 72 of 74 patients (97%) with no significant difference between IDUs and nonaddicts (Table 3). TEE was performed significantly more often in nonaddicts than in IDUs (74% vs 45%).

A vegetation was evident by echocardiography in 53% of cases and a new regurgitation in 71% of cases without significant differences between the groups (Table 3). Only 4 patients had an intracardial abscess or valve perforation.

### Clinical manifestations and outcome

There were no differences in the clinical manifestations between the groups except the tendency for more frequent occurrence of various vascular phenomena among IDUs (Table 4). The most common infection focus was skin or soft tissue infection in 46 of 74 patients (62%). An extracardiac deep infection was found in 85% of IDUs and in 89% of nonaddicts (*P *= 0.70).

**Table 4:** Clinical manifestations of injection drug users and nonaddicts with *Staphylococcus aureus *endocarditis during 3 months follow-up.

	Injection drug users	Nonaddicts	Total			
Variable	(*n *= 20)	(*n *= 54)	(*n *= 74)	OR	95% CI	*P*
Skin or soft tissue	12 (60)	34 (63)	46 (62)	0.88	0.31–2.53	1.00
Central intravenous catheter	0 (0)	3 (6)	3 (4)	-	-	0.56
Extracardiac deep infection^a^	17 (85)	48 (89)	65 (88)	0.71	0.16–3.15	0.70
Pneumonia	14 (70)	27 (50)	41 (55)	2.33	0.78–6.98	0.19
Deep-seated abscess^b^	7 (35)	25 (46)	32 (43)	0.63	0.22–1.81	0.44
Osteomyelitis	6 (30)	17 (32)	23 (31)	0.93	0.31–2.85	1.00
Septic arthritis	3 (15)	5 (9)	8 (11)	1.73	0.37–8.02	0.67
Urinary tract	1 (5)	4 (7)	5 (7)	0.66	0.07–6.27	1.00
Recurrent *S. aureus *bacteremia	1 (5)	1 (2)	2 (3)	2.79	0.17–46.84	0.47
Mediastinitis	0 (0)	9 (17)	9 (12)	-	-	0.10
Vascular phenomenon	12 (60)	19 (35)	31 (42)	2.76	0.96–7.93	0.07
Arterial thromboembolic event	5 (25)	17 (32)	22 (30)	0.73	0.23–2.32	0.78
Heart^c^	0 (0)	11 (20)	11 (15)	-	-	0.03
Spleen, kidney or liver	3 (15)	0 (0)	3 (4)	-	-	0.02
Cerebral	4 (20)	9 (17)	13 (18)	1.25	0.34–4.63	0.74
Venous thromboembolic event	8 (40)	4 (7)	12 (16)	8.33	2.15–32.32	0.002
Pulmonary embolism^d^	8 (40)	2 (4)	10 (14)	17.33	3.26–92.24	<0.001
Deep venous thrombosis	0 (0)	2 (4)	2 (3)	-	-	1.00

**NOTE. **Data are no. (%) of patients, unless otherwise indicated.

^a ^Each patient has been included once although some patients had several extracardiac deep infections at various time points.

^b ^Intramuscular, epidural, cerebral, parenchymal, lung, peritoneal, subphrenic, gynecological and pericardial abscesses, or pleural empyema.

^c ^Acute myocardial infarction or unstable angina.

^d ^Includes 8 patients with septic pulmonary embolism and 2 patients with venous pulmonary embolism.

Vascular complications, including arterial or venous thromboembolic events, were detected in 60% of IDUs but in only 35% of nonaddicts (*P *= 0.07) (Table 4). Especially, septic pulmonary embolism was observed only in IDUs. Whereas all coronary artery related diseases were among nonaddicts, all 3 parenchymal embolic events were observed in drug abusers. Congestive heart failure in acute phase was present in 11 of 74 patients (15%) with no difference between the groups. Two patients developed recurrent bacteremia during the 3 months follow-up period. There were no differences in median duration of fever (3 days) or median duration of hospitalization (32 days) between IDUs and nonaddicts (Table 5). Cardiac surgery was performed in 15% of IDUs but only in 7% of nonaddicts (OR, 2.21; 95% CI, 0.45–10.87; *P *= 0.38).

**Table 5:** Outcome and surgical treatment of injection drug users and nonaddicts with *Staphylococcus aureus *endocarditis.

	Injection drug users	Nonaddicts	Total	
Outcome	(*n *= 20)	(*n *= 54)	(*n *= 74)	*P*
Case fatality rate				
At 7 days	1 (5)	6 (11)	7 (9)	0.67
At 28 days	1 (5)	16 (30)	17 (23)	0.03
At 3 months	2 (10)	21 (39)	23 (31)	0.02
Duration of fever, median days (IQR)^a^	3 (2–10)	3 (0–6)	3 (1–6)	0.24
Duration of hospitalization, median days (IQR)	32 (30–48)	32 (20–47)	32 (24–47)	0.55
Cardiac surgery	3 (15)	4 (7)	7 (9)	0.38
Valve replacement	1 (5)	4 (7)	5 (7)	1.00
Valve repair^b^	3 (15)	0 (0)	3 (4)	0.02

**NOTE. **Data are no. (%) of patients, unless otherwise indicated. IQR, interquartile range.

^a ^Fever > 37.5°C after the first positive blood culture for *S. aureus *with 72 patients (2 patients excluded, death before defervescence).

^b ^Includes 2 patients with vegetectomy and 1 patient with annuloplasty.

Case fatality rate of all patients with IE was 23% at 28 days, and 31% at 3 months (Table 5). Mortality was significantly higher in nonaddicts than in addicts at 28 days (OR, 8.00; 95% CI, 0.99–64.94; *P *= 0.03), and also at 3 months (OR, 5.73; 95% CI, 1.20–27.25; *P *= 0.02). Significant factors for lower mortality based on univariate analyses were injection drug abuse (HR, 0.22; 95% CI, 0.05–0.92; *P *= 0.04), age (HR, 1.03; 95% CI, 1.01–1.06; *P *= 0.006), and none or nonfatal underlying diseases by McCabe's classification (HR, 0.24; 95% CI, 0.10–0.54; *P *= 0.001). Statistically significant association for mortality were not found with the following: right-sided involvement (HR, 0.30; 95% CI, 0.07–1.30; *P *= 0.11), left-sided involvement (HR, 2.31; 95% CI, 0.69–7.78; *P *= 0.18), severe sepsis at the time of first positive blood culture for *S. aureus *(HR, 1.71; 95% CI, 0.74–3.95; *P *= 0.21), or arterial embolic events (HR, 2.15; 95% CI, 0.94–4.90; *P *= 0.07). After adjusting by age and underlying diseases, injection drug abuse was not significantly associated with lower mortality (HR, 0.74; 95% CI, 0.10–5.40; *P *= 0.77).

### Laboratory data

At the time of first positive blood culture for *S. aureus*, leucocytosis was significantly more common in nonaddicts than in IDUs (55% vs 15%, respectively; *P *= 0.003), and only 2 patients (4%) had leucopenia. The median of serum C-reactive protein on the day of the first positive blood culture for *S. aureus *was 198 mg/L (range, 60–413 mg/L) in addicts compared with 171 mg/L (range, 5–478 mg/L) in nonaddicts without significant difference between the groups. Only 2 nonaddicts and none of IDUs had alanine aminotransferase level 2-fold above normal limit.

### Antibiotic treatment

Sixty-five of 74 patients (88%) were treated with cloxacillin or dicloxacillin with no significant difference between the groups. Seven patients (10%) received cefuroxime, and one received vancomycin and one ceftriaxone. The median duration of parenteral antibiotic therapy from the first positive blood culture for *S. aureus *was 30 days (interquartile range, 24–43 days) in IDUs and 26 days (interquartile range, 13–34 days) in nonaddicts (*P *= 0.12), respectively.

An aminoglycoside was significantly more often given to addicts (19 of 20 patients) than to nonaddicts (29 of 54 patients) (OR, 16.38; 95% CI, 2.05–131.21; *P *= 0.001). Eighteen of 20 IDUs (90%) received rifampicin compared with 48 of 54 nonaddicts (89%) with no significant difference between the groups.

## Discussion

To the best of our knowledge, this trial is one of the largest prospective evaluations on *S. aureus *endocarditis comparing patients with and without injection drug use [2,13]. It is to be noted that our trial included only methicillin-sensitive *S. aureus *(MSSA) strains. MRSA strains are very rare in Finland (< 2% from all isolated *S. aureus *strains) and not a single endocarditis due to MRSA had to be excluded. Lack of MRSA might complicate extrapolation of these results into countries with high MRSA prevalence. However, MRSA endocarditis is usually more difficult to treat. While concentrating only on cases caused by MSSA strains real differences between IDUs and nonaddicts might be better revealed. In our trial, right-sided involvement predominated slightly in IDUs whereas almost all nonaddicts had left-sided endocarditis. In contrast to previous data the frequency of complications among IDUs was equal to that among nonaddicts. An extracardiac deep infection was observed also in most of the addicts. According to recent studies, deep infections seem to be more common in SAB than previously thought [20,25] [see Additional file 1]. In the current trial, the frequency of arterial embolic events was similar in both groups, although they have been observed less frequently among IDUs in earlier studies [13,18,26]. However, mortality in addicts was significantly lower than in nonaddicts as reported previously [13,14,26,27].

*S. aureus *is an important and a more common cause of IE, especially among IDUs [8,11]. In a recent prospective multicenter study of 505 patients with SAB, endocarditis was found in 13% of all patients and in 35% of IDUs [6]. In agreement with that study, the incidence of IE was 17% among all SAB patients and 46% among IDUs in our trial. The proportion of definite IE in the present trial (76%) was also in line that observed in the other large survey [6].

Frequent use of echocardiography may increase the incidence of IE at least to some extent. Persistent bacteremia for over 3 days has been suggested to be a predictor for endocarditis [28] which was not designed for evaluation in our trial. We performed echocardiography as clinically indicated. It was done to 61% of SAB patients, and the rate was similar or higher compared with other studies [19,25,29]. Some endocarditis cases with atypical presentation might have been missed because echocardiography in our cohort as well as in previous studies was not performed for all SAB patients. The tricuspid valve was predominantly affected in addicts (60%), although the incidence was slightly lower than that reported previously (from 70% to 90%) [2,13,19,30]. We presented that the frequency of left-sided involvement in addicts was as high as 30% which is similar to that in some studies [2,10,31], but higher than in most earlier reports with left-sided endocarditis ranging from 8% to 19% [16,26,30]. In IDUs, both sides of the heart are usually involved simultaneously in 5% to 10% of cases as detected in the current trial.

Extracardiac deep infections have been associated with SAB in left-sided endocarditis with the incidence ranging from 40% to 76% [20,32-34] [see Additional file 1]. In contrast to previous studies [13,26,35], deep infections in our trial occurred in over 80% in both IDUs and nonaddicts. Systemic thromboembolic events are well recognized complications in endocarditis occurring in 21% to 50% of cases, especially in patients with left-sided involvement and prosthetic heart valves, and are accompanied by high mortality rates [36-39]. In earlier reports, IDUs had fewer arterial emboli or strokes than nonaddicts probably due to involvement of right-sided endocarditis [2,18,40]. In contrast, in our trial arterial thromboembolic events were observed with equal frequency in addicts and in nonaddicts. Venous embolic events were more common among addicts, and septic pulmonary embolism manifestated only in IDUs. However, the frequency of septic pulmonary embolism (40%) was less than the previously reported incidence of 67% to 87% in IDUs [40-42].

Due to lack of controlled studies, indications for surgical approaches are not determined in patients with drug addiction [27]. Furthermore, doubts on compliance and continued drug abuse may reduce enthusiasm for surgery. Cardiac surgery is thought to be needed in only a small minority of cases in IDUs, and indications for surgical correction of right-sided involvement are not equally defined as for left-sided endocarditis [16,41,43]. However, some studies suggest that surgical treatment may clearly improve survival in IE among IDUs [10,44]. In this trial, cardiac surgery was as common in IDUs as in nonaddicts (15% vs 7%), but valve repair was performed more often in addicts. The frequency of cardiac surgery in IDUs was high in light of prosthetic valve domination in nonaddicts in agreement with another recent study [2].

The duration of parenteral antibiotic treatment in SAB depends largely on the presence of an associated IE, in which 4 to 6 weeks therapy is recommended [15,45,46]. A shorter 2 weeks treatment has been suggested for selected cases of right-sided involvement with a good prognosis [17,45]. Recommendations on duration of aminoglycoside are variable. For left-sided native valve endocarditis 3–5 days therapy is suggested but as long as 2 weeks for right-sided involvement has been used [47,48]. In addition, two small studies have demonstrated oral fluoroquinolone and rifampicin for 4 weeks to be as effective as the standard intravenous therapy in right-sided endocarditis [49,50]. In our cohort, the high proportion of extracardiac deep infections in addicts extended the intravenous antibiotic therapy to 4 weeks. This observation suggest that deep infections should be actively searched for also among IDUs.

This trial was not designed to reveal possible differences between antibiotic treatments and the statistical power would neither enable such analysis. However, in accordance with positive results in experimental and animal studies we found lower mortality in SAB with a deep infection when the patients were treated with rifampicin in addition to standard treatment [20] [see Additional file 1]. In the present trial, most IDUs were also treated with rifampicin which might cause problems with drug interactions not least with methadone.

Overall mortality rate was significantly higher among nonaddicts (39%) than in addicts (10%) in accordance with 30% to 70% mortality previously reported in nonaddicts [1,5,14]. In earlier studies, poor prognosis has been associated with left-sided involvement, endocarditis of prosthetic valve, higher incidence of underlying diseases, central nervous system manifestations, and older age [1,14,19,29]. Our trial showed that injection drug abuse in accordance with younger age and lack of severe underlying diseases were associated with better prognosis. There was a tendency for lower mortality in patients with right-sided IE but for higher mortality in patients with severe sepsis, left-sided involvement, and arterial embolic events. However, these factors did not achieve statistical significance probably due to insufficient sample size.

## Conclusion

We concluded that endocarditis was more frequently connected to SAB among IDUs. In contrast to previous reports, addicts had equally often complications including extracardiac deep infections and arterial thromboembolic events as nonaddicts. In spite of this, mortality was significantly lower in IDUs in accordance with earlier data.

## Competing interests

Financial competing interests: ER has served as consultant to Aventis Pharma. AJ and VV have been consultants to several companies including Aventis Pharma, Bayer, Pfizer Inc., AstraZeneca, Roche, Pharmacia and Merck & Co, Inc. None of the authors had non-financial competing interests to disclose.

## Authors' contributions

Authors ER, KS, JL, VV, and AJ participated in the design and coordination of the study. ER, KS, and JL made substantial contributions to acquisition of data. ER, KH, SS, VV, and AJ performed statistical analysis. All authors read and approved the final manuscript.

## Pre-publication history

The pre-publication history for this paper can be accessed here:



## Supplementary Material

Additional file 1 **Levofloxacin does not decrease mortality in *Staphylococcus aureus *bacteraemia when added to the standard treatment: A prospective and randomized clinical trial of 381 patients**. This article represents original trial where the effect of levofloxacin in addition to standard antistaphylococcal treatment of SAB was studied in relation to patient outcome and development of complications. Click here for file

## References

[B1] Miro JM, Anguera I, Cabell CH, Chen AY, Stafford JA, Corey GR, Olaison L, Eykyn S, Hoen B, Abrutyn E, Raoult D, Bayer A, Fowler VGJ (2005). Staphylococcus aureus native valve infective endocarditis: report of 566 episodes from the International Collaboration on Endocarditis Merged Database. Clin Infect Dis.

[B2] Fowler VGJ, Miro JM, Hoen B, Cabell CH, Abrutyn E, Rubinstein E, Corey GR, Spelman D, Bradley SF, Barsic B, Pappas PA, Anstrom KJ, Wray D, Fortes CQ, Anguera I, Athan E, Jones P, van der Meer JT, Elliott TS, Levine DP, Bayer AS (2005). Staphylococcus aureus endocarditis: a consequence of medical progress. Jama.

[B3] Mylonakis E, Calderwood SB (2001). Infective endocarditis in adults. N Engl J Med.

[B4] Mirimanoff RO, Glauser MP (1982). Endocarditis during Staphylococcus aureus septicemia in a population of non-drug addicts. Arch Intern Med.

[B5] Hedstrom SA, Christensson B (1983). Staphylococcus aureus septicaemia and endocarditis at the University Hospital in Lund 1976-1980. Scand J Infect Dis Suppl.

[B6] Chang FY, MacDonald BB, Peacock JEJ, Musher DM, Triplett P, Mylotte JM, O'Donnell A, Wagener MM, Yu VL (2003). A prospective multicenter study of Staphylococcus aureus bacteremia: incidence of endocarditis, risk factors for mortality, and clinical impact of methicillin resistance. Medicine (Baltimore).

[B7] Cecchi E, Forno D, Imazio M, Migliardi A, Gnavi R, Dal Conte I, Trinchero R (2004). New trends in the epidemiological and clinical features of infective endocarditis: results of a multicenter prospective study. Ital Heart J.

[B8] Moreillon P, Que YA (2004). Infective endocarditis. Lancet.

[B9] Bassetti S, Battegay M (2004). Staphylococcus aureus infections in injection drug users: risk factors and prevention strategies. Infection.

[B10] Mathew J, Addai T, Anand A, Morrobel A, Maheshwari P, Freels S (1995). Clinical features, site of involvement, bacteriologic findings, and outcome of infective endocarditis in intravenous drug users. Arch Intern Med.

[B11] Wilson LE, Thomas DL, Astemborski J, Freedman TL, Vlahov D (2002). Prospective study of infective endocarditis among injection drug users. J Infect Dis.

[B12] Gordon RJ, Lowy FD (2005). Bacterial infections in drug users. N Engl J Med.

[B13] Chambers HF, Korzeniowski OM, Sande MA (1983). Staphylococcus aureus endocarditis: clinical manifestations in addicts and nonaddicts. Medicine (Baltimore).

[B14] Julander I (1985). Unfavourable prognostic factors in Staphylococcus aureus septicemia and endocarditis. Scand J Infect Dis.

[B15] Karchmer AW, Braunwald E, Zipes DP, Libby P (2001). Infective endocarditis. Heart Disease: A Textbook of Cardiovascular Medicine.

[B16] Miro JM, del Rio A, Mestres CA (2003). Infective endocarditis and cardiac surgery in intravenous drug abusers and HIV-1 infected patients. Cardiol Clin.

[B17] Torres-Tortosa M, de Cueto M, Vergara A, Sanchez-Porto A, Perez-Guzman E, Gonzalez-Serrano M, Canueto J (1994). Prospective evaluation of a two-week course of intravenous antibiotics in intravenous drug addicts with infective endocarditis. Grupo de Estudio de Enfermedades Infecciosas de la Provincia de Cadiz. Eur J Clin Microbiol Infect Dis.

[B18] Hart RG, Foster JW, Luther MF, Kanter MC (1990). Stroke in infective endocarditis. Stroke.

[B19] Watanakunakorn C (1994). Staphylococcus aureus endocarditis at a community teaching hospital, 1980 to 1991. An analysis of 106 cases. Arch Intern Med.

[B20] Ruotsalainen E, Järvinen A, Koivula I, Kauma H, Rintala E, Lumio J, Kotilainen P, Vaara M, Nikoskelainen J, Valtonen V (2006). Levofloxacin does not decrease mortality in Staphylococcus aureus bacteraemia when added to the standard treatment: a prospective and randomized clinical trial of 381 patients. J Intern Med.

[B21] Li JS, Sexton DJ, Mick N, Nettles R, Fowler VGJ, Ryan T, Bashore T, Corey GR (2000). Proposed modifications to the Duke criteria for the diagnosis of infective endocarditis. Clin Infect Dis.

[B22] McCabe WR, Jackson GG (1962). Gram negative bacteraemia 1. Etiology and ecology. Arch Intern Med.

[B23] Levy MM, Fink MP, Marshall JC, Abraham E, Angus D, Cook D, Cohen J, Opal SM, Vincent JL, Ramsay G (2003). 2001 SCCM/ESICM/ACCP/ATS/SIS International Sepsis Definitions Conference. Crit Care Med.

[B24] Mermel LA, Farr BM, Sherertz RJ, Raad II, O'Grady N, Harris JS, Craven DE (2001). Guidelines for the management of intravascular catheter-related infections. Clin Infect Dis.

[B25] Fowler VGJ, Olsen MK, Corey GR, Woods CW, Cabell CH, Reller LB, Cheng AC, Dudley T, Oddone EZ (2003). Clinical identifiers of complicated Staphylococcus aureus bacteremia. Arch Intern Med.

[B26] Levine DP, Crane LR, Zervos MJ (1986). Bacteremia in narcotic addicts at the Detroit Medical Center. II. Infectious endocarditis: a prospective comparative study. Rev Infect Dis.

[B27] Moss R, Munt B (2003). Injection drug use and right sided endocarditis. Heart.

[B28] Khatib R, Johnson LB, Fakih MG, Riederer K, Khosrovaneh A, Shamse Tabriz M, Sharma M, Saeed S (2006). Persistence in Staphylococcus aureus bacteremia: Incidence, characteristics of patients and outcome. Scand J Infect Dis.

[B29] Mylotte JM, Tayara A (2000). Staphylococcus aureus bacteremia: predictors of 30-day mortality in a large cohort. Clin Infect Dis.

[B30] Julander I (1983). Staphylococcal septicaemia and endocarditis in 80 drug addicts. Aspects on epidemiology, clinical and laboratory findings and prognosis. Scand J Infect Dis Suppl.

[B31] Graves MK, Soto L (1992). Left-sided endocarditis in parenteral drug abusers: recent experience at a large community hospital. South Med J.

[B32] Bayer AS, Lam K, Ginzton L, Norman DC, Chiu CY, Ward JI (1987). Staphylococcus aureus bacteremia. Clinical, serologic, and echocardiographic findings in patients with and without endocarditis. Arch Intern Med.

[B33] Fowler VGJ, Schelenz S, Bayer AS, Mandell GL, Bennett JE, Dolin R (2005). Endocarditis and intravascular infections. Mandell, Douglas and Bennett`s Principles and Practice of Infectious Diseases Volume 2.

[B34] Fowler VGJ, Sanders LL, Kong LK, McClelland RS, Gottlieb GS, Li J, Ryan T, Sexton DJ, Roussakis G, Harrell LJ, Corey GR (1999). Infective endocarditis due to Staphylococcus aureus: 59 prospectively identified cases with follow-up. Clin Infect Dis.

[B35] Ringberg H, Thoren A, Lilja B (2000). Metastatic complications of Staphylococcus aureus septicemia. To seek is to find. Infection.

[B36] Baddour LM, Bisno AL (1986). Infective endocarditis complicating mitral valve prolapse: epidemiologic, clinical, and microbiologic aspects. Rev Infect Dis.

[B37] Fabri JJ, Issa VS, Pomerantzeff PM, Grinberg M, Barretto AC, Mansur AJ (2006). Time-related distribution, risk factors and prognostic influence of embolism in patients with left-sided infective endocarditis. Int J Cardiol.

[B38] Hasbun R, Vikram HR, Barakat LA, Buenconsejo J, Quagliarello VJ (2003). Complicated left-sided native valve endocarditis in adults: risk classification for mortality. JAMA.

[B39] Thuny F, Disalvo G, Belliard O, Avierinos JF, Pergola V, Rosenberg V, Casalta JP, Gouvernet J, Derumeaux G, Iarussi D, Ambrosi P, Calabro R, Riberi A, Collart F, Metras D, Lepidi H, Raoult D, Harle JR, Weiller PJ, Cohen A, Habib G (2005). Risk of embolism and death in infective endocarditis: prognostic value of echocardiography: a prospective multicenter study. Circulation.

[B40] Hecht SR, Berger M (1992). Right-sided endocarditis in intravenous drug users. Prognostic features in 102 episodes. Ann Intern Med.

[B41] Bouza E, Menasalvas A, Munoz P, Vasallo FJ, del Mar Moreno M, Garcia Fernandez MA (2001). Infective endocarditis-a prospective study at the end of the twentieth century: new predisposing conditions, new etiologic agents, and still a high mortality. Medicine (Baltimore).

[B42] Sklaver AR, Hoffman TA, Greenman RL (1978). Staphylococcal endocarditis in addicts. South Med J.

[B43] Faber M, Frimodt-Moller N, Espersen F, Skinhoj P, Rosdahl V (1995). Staphylococcus aureus endocarditis in Danish intravenous drug users: high proportion of left-sided endocarditis. Scand J Infect Dis.

[B44] Arbulu A, Holmes RJ, Asfaw I (1993). Surgical treatment of intractable right-sided infective endocarditis in drug addicts: 25 years experience. J Heart Valve Dis.

[B45] Baddour LM, Wilson WR, Bayer AS, Fowler VGJ, Bolger AF, Levison ME, Ferrieri P, Gerber MA, Tani LY, Gewitz MH, Tong DC, Steckelberg JM, Baltimore RS, Shulman ST, Burns JC, Falace DA, Newburger JW, Pallasch TJ, Takahashi M, Taubert KA (2005). Infective endocarditis: diagnosis, antimicrobial therapy, and management of complications: a statement for healthcare professionals from the Committee on Rheumatic Fever, Endocarditis, and Kawasaki Disease, Council on Cardiovascular Disease in the Young, and the Councils on Clinical Cardiology, Stroke, and Cardiovascular Surgery and Anesthesia, American Heart Association: endorsed by the Infectious Diseases Society of America. Circulation.

[B46] Moreillon P, Que YA, Glauser MP, Mandell GL, Bennett JE, Dolin R (2005). Staphylococcus aureus (including staphylococcal toxic shock). Mandell, Douglas, and Bennett`s Principles and Practice of Infectious Diseases Volume 2.

[B47] Lowy FD (1998). Staphylococcus aureus infections. N Engl J Med.

[B48] Petti CA, Fowler VGJ (2002). Staphylococcus aureus bacteremia and endocarditis. Infect Dis Clin North Am.

[B49] Heldman AW, Hartert TV, Ray SC, Daoud EG, Kowalski TE, Pompili VJ, Sisson SD, Tidmore WC, vom Eigen KA, Goodman SN, Lietman PS, Petty BG, Flexner C (1996). Oral antibiotic treatment of right-sided staphylococcal endocarditis in injection drug users: prospective randomized comparison with parenteral therapy. Am J Med.

[B50] Dworkin RJ, Lee BL, Sande MA, Chambers HF (1989). Treatment of right-sided Staphylococcus aureus endocarditis in intravenous drug users with ciprofloxacin and rifampicin. Lancet.

