# Supplementary material for: Clinical manifestations and outcome in Staphylococcus aureus endocarditis among injection drug users and nonaddicts: a prospective study of 74 patients
Source: BMC Infect Dis. 2006 Sep 11;6:137. doi: 10.1186/1471-2334-6-137 (PMC1584240; doi:10.1186/1471-2334-6-137)
Supplement: Additional file 1 — Levofloxacin does not decrease mortality in Staphylococcus aureus bacteraemia when added to the standard treatment: A prospective and randomized clinical trial of 381 patients. This article represents original trial where the effect of levofloxacin in addition to standard antistaphylococcal treatment of SAB was studied in relation to patient outcome and development of complications. [file 1471-2334-6-137-S1.pdf]

Journal of Internal Medicine 2006; **259**: 179–190 doi:10.1111/j.1365-2796.2005.01598.x

Levofloxacin does not decrease mortality in *Staphylococcus aureus* bacteraemia when added to the standard treatment: a prospective and randomized clinical trial of 381 patients

E. RUOTSALAINEN[1], A. JÄRVINEN[1], I. KOIVULA[2], H. KAUMA[3], E. RINTALA[4], J. LUMIO[5], P. KOTILAINEN[6], M. VAARA[7], J. NIKOSKELAINEN[6], V. VALTONEN[1] & THE FINLEVO STUDY GROUP*

From the [1]Division of Infectious Diseases, Department of Medicine, Helsinki University Central Hospital, Helsinki; [2]Department of Medicine, Kuopio University Hospital, Kuopio; [3]Division of Infectious Diseases, Department of Medicine, Oulu University Hospital, Oulu; [4]Department of Medicine, Satakunta Central Hospital, Pori; [5]Department of Medicine, Tampere University Hospital, Tampere; [6]Department of Medicine, Turku University Central Hospital, Turku; and [7]Division of Clinical Microbiology, Helsinki University Central Hospital Laboratory, Helsinki; Finland

Abstract. Ruotsalainen E, Järvinen A, Koivula I, Kauma H, Rintala E, Lumio J, Kotilainen P, Vaara M, Nikoskelainen J, Valtonen V, the FINLEVO Study Group (Helsinki University Central Hospital, Helsinki; Kuopio University Hospital, Kuopio; Oulu University Hospital, Oulu; Satakunta Central Hospital, Pori; Tampere University Hospital, Tampere; Turku University Central Hospital, Turku; and Helsinki University Central Hospital Laboratory, Helsinki; Finland). Levofloxacin does not decrease mortality in *Staphylococcus aureus* bacteraemia when added to the standard treatment: a prospective and randomized clinical trial of 381 patients. *J Intern Med* 2006; **259**: 179–190.

Objectives. To study whether levofloxacin, added to standard treatment, could reduce the high mortality and complication rates in *Staphylococcus aureus* bacteraemia.

Design. A prospective randomized multicentre trial from January 2000 to August 2002.

Setting. Thirteen tertiary care or university hospitals in Finland.

Subjects. Three hundred and eighty-one adult patients with *S. aureus* bacteraemia. Patients with meningitis, and those with fluoroquinolone- or methicillin-resistant *S. aureus* were excluded.

Interventions. Standard treatment (mostly semisynthetic penicillin) ($n = 190$) or that combined with levofloxacin ($n = 191$). Supplementary rifampicin was recommended if deep infection was suspected.

Main outcome measures. Primary end-points were mortality at 28 days and at 3 months. Clinical and laboratory parameters were analysed as secondary end-points.

Results. Adding levofloxacin to the standard treatment offered no survival benefit. Case fatality rates were 14% in both groups at 28 days, and 21% in the standard treatment and 18% in the levofloxacin group at 3 months. Levofloxacin combination did not differ from the standard treatment in the number of complications, time to defervescence, decrease in serum C-reactive protein concentration or length of antibiotic treatment. Deep infection was found in 84% of patients within 1 week following randomization with no difference between the treatment groups. At 3 months, the case fatality rate for patients with deep infection was 17% amongst those who received rifampicin versus 38% for those without rifampicin

*The FINLEVO Study Group is composed of the authors and the following investigators: Pentti Kuusela, MD, Division of Clinical Microbiology, Helsinki University Central Hospital Laboratory, Helsinki; Eija Ruotsalainen, MD, and Ulla Kärkkäinen, MD, Kuopio University Hospital, Kuopio; Janne Laine, MD, Risto Vuento, MD, and Jaana Syrjänen, MD, Tampere University Hospital, Tampere; Pekka Ylipalosaari, MD, Pekka Kujala, MD, Markku Koskela, MD, and Hannu Syrjälä, MD, Oulu University Hospital, Oulu; Olli Meurman, MD, and Ulla Hohenthal, MD, Turku University Central Hospital, Turku; Raija Uusitalo-Seppälä, MD, and Sini Pajarre, MD, Satakunta Central Hospital, Pori; Kari Sammalkorpi, MD, Maria Hospital, Helsinki; Kirsti Sandberg, MD, Peijas Hospital, Vantaa; Ilpo Simula, MD, and Anja Kostiala-Thompson, MD, Jorvi Hospital, Espoo; Markku Broas, MD, Outi Kiviniemi, MD, and Henrik Jägerroos, MD, Lapland Central Hospital, Rovaniemi; Jukka Heikkinen, MD, and Suvi-Sirkku Kaukoranta-Tolvanen, MD, North Karelian Central Hospital, Joensuu.

© 2005 Blackwell Publishing Ltd

($P < 0.001$, odds ratio = 3.06, 95% confidence intervals = 1.69–5.54).
Conclusions. Levofloxacin combined with standard treatment in *S. aureus* bacteraemia did not decrease mortality or the incidence of deep infections, nor did it speed up recovery. Interestingly, deep infections in *S. aureus* bacteraemia appeared to be more common than previously reported.

Keywords: bacteraemia, fluoroquinolone, levofloxacin, rifampicin, sepsis, *Staphylococcus aureus*.

Introduction

Staphylococcus aureus is the second most common bloodstream isolate both in hospital and community-acquired bacteraemias in all age groups. *Staphylococcus aureus* bacteraemia (SAB) still confers remarkably high mortality, ranging up to 60% in some studies, although antistaphylococcal antibiotics have been available for more than 40 years [1–4]. During recent decades there has been no significant improvement in the outcome of staphylococcal infections, which cannot be entirely explained by the increased incidence of methicillin-resistant *S. aureus* (MRSA) [5, 6]. The clinical course of SAB is determined by its complications, particularly by the development of deep infections due to metastatic spread, and by the high recurrence rate of bacteraemia [6–12]. The reported frequency of metastatic complications varies greatly, from 10% to 50% [9, 13–16].

The standard treatment strategy for bacteraemic methicillin-sensitive *S. aureus* infections includes a semisynthetic penicillin, such as nafcillin, cloxacillin or dicloxacillin. In endocarditis, an aminoglycoside for 3–14 days is combined with standard treatment [12, 17, 18]. Experimental studies and some small clinical trials suggest that adding rifampicin could improve treatment results in deep infections [19–21], but recommendations on its use are variable [12, 18, 22–24].

Levofloxacin is a fluoroquinolone with improved activity against Gram-positive bacteria including *S. aureus in vitro* [25]. In experimental studies fluoroquinolones have shown an additive effect in combination with standard antistaphylococcal therapy in severe *S. aureus* infections [26]. Furthermore, fluoroquinolones have been combined with rifampicin to provide an entirely oral regimen in staphylococcal right-sided endocarditis [27, 28], chronic osteomyelitis or foreign body infections [29, 30] and other deep-seated abscesses [31]. In SAB, most deep infections are observed within 2 weeks after the onset of bacteraemia [32]. The metastatic complications might be prevented by early treatment with bactericidal fluoroquinolone, which penetrates well into tissues.

We here report the results of the first prospective trial where the effect of levofloxacin in addition to standard antistaphylococcal treatment of SAB was studied in relation to patient outcome and development of complications.

Patients and methods

Study design

This was a prospective, randomized, multicentre trial conducted in five university hospitals and seven tertiary care hospitals in Finland. Adult patients with at least one blood culture positive for *S. aureus* were included within 1–7 days of blood culture sampling from January 2000 through to August 2002. Randomization was done blindly and separately at each study location after the patient or his/her representative had given written informed consent. After randomization, the treatments were open for the investigator and the patient. The trial was approved by the ethics committees of all study sites and by the Finnish National Agency for Medicines.

Exclusion criteria included age younger than 18 years, imprisonment, proven or suspected pregnancy, breastfeeding, epilepsy, another bacteraemia during the previous 28 days, polymicrobial bacteraemia (≥3 microbes), history of allergy to any quinolone antibiotic, previous tendinitis during fluoroquinolone therapy, prior fluoroquinolone use for more than 5 days before randomization, positive culture for *S. aureus* only from a central intravenous catheter, neutropenia ($<0.5 \times 10^9$ L^{-1}) or failure to supply an informed consent. Patients with bacteraemia due to MRSA and a *S. aureus* strain resistant

to any fluoroquinolone, and those with meningitis at the time of randomization, were also excluded.

Study treatments

Patients with SAB were randomly assigned to receive either standard treatment or standard treatment combined with levofloxacin. The dose of levofloxacin was 500 mg once daily for patients under 60 kg and 500 mg b.i.d. for those over 60 kg in weight, both intravenously and orally. Primarily, the standard treatment consisted of a semisynthetic penicillin, cloxacillin or dicloxacillin (2 g q 4 h), intravenously. Alternatively, cefuroxime (1.5 g q 6 h), clindamycin (600 mg q 6–8 h), or vancomycin (1 g b.i.d.) were allowed if a contraindication against the use of penicillins was noted. When oral treatment was indicated, cloxacillin (500 mg q 6 h), cephalexin or cefadroxil (500 mg q 6 h), or clindamycin (300 mg q 6 h) were accepted as standard therapy. In cases of renal dysfunction, the antibiotic doses were adjusted as recommended by the manufacturers.

If endocarditis was clinically suspected or confirmed, aminoglycoside (either tobramycin or netilmicin at 1 mg per kilogram of body weight q 8 h) was added to the drug therapy described above. Rifampicin (450 mg once daily for patients under 50 and 600 mg once daily for patients over 50 kg in weight, orally or intravenously) was given if there was a suspicion or evidence of endocarditis or other deep infections such as pneumonia, deep-seated abscess, osteomyelitis, septic arthritis, mediastinitis or infection of a prosthetic device.

The duration of antibiotic treatment was determined by the treating doctor. However, all patients received at least 14 days of intravenous antibiotic treatment. In SAB associated with a central intravenous catheter the antibiotic treatment was discontinued after 14 days when the catheter was replaced [33]. Parenteral antibiotic therapy was switched to oral dosing after 14 days in patients with no signs of a deep infection, if the serum C-reactive protein (CRP) concentration was <10 mg L^{-1} and the patient was afebrile [13, 16, 34]. When a deep infection was verified or clinically suspected, intravenous antibiotic treatment and rifampicin were recommended to be continued for at least 28 days. In cases of endocarditis, aminoglycoside treatment was discontinued after 7 days [12, 35].

Definitions

Staphylococcus aureus bacteraemia was hospital-acquired if the first positive blood culture was obtained ≥48 h after admission, or the patient was a resident in a long-term care facility or attended haemodialysis within the preceding 2 months. Prognosis or severity of underlying diseases were classified as healthy, nonfatal, ultimately or rapidly fatal according to the criteria of McCabe and Jackson [36].

The infection focus was defined as definite if it was documented by bacteriological, radiological or pathological investigations, but suspected if it was evident from clinical findings only. Infection of a central intravenous catheter was defined by the guidelines of the Infectious Diseases Society of America [33]. Endocarditis was classified as definite or possible using the modified Duke criteria [37]. Relapse of SAB was confirmed by the same resistance pattern and pulsed-field gel electrophoresis typing for two *S. aureus* strains. Other recurrences of *S. aureus* culture in the blood were classified as reinfections.

End-points

All patients were followed up by an infectious disease specialist during the hospital treatment and thereafter with control visits at 28 days and at 3 months. Primary end-points were case fatality rate at 28 days and at 3 months. Secondary outcome measures were the number of complications (e.g. deep infections) observed after the first week, decrease in serum CRP concentration, length of antibiotic treatment, need for surgical intervention, and time to defervescence (recorded in days until axillary temperature was <37.5 °C). Laboratory tests were conducted on the day of positive blood culture for *S. aureus*, at randomization and every other day during the first week, twice a week thereafter during hospitalization, at 28 days, and at 3 months.

Sample size and statistical analysis

In the sample size calculation, when mortality was assumed to be 10% in the levofloxacin group and 20% in the standard treatment group, a power of 80% would be achieved with 198 patients in each study arm. A two-tailed significance level of 5% was used.

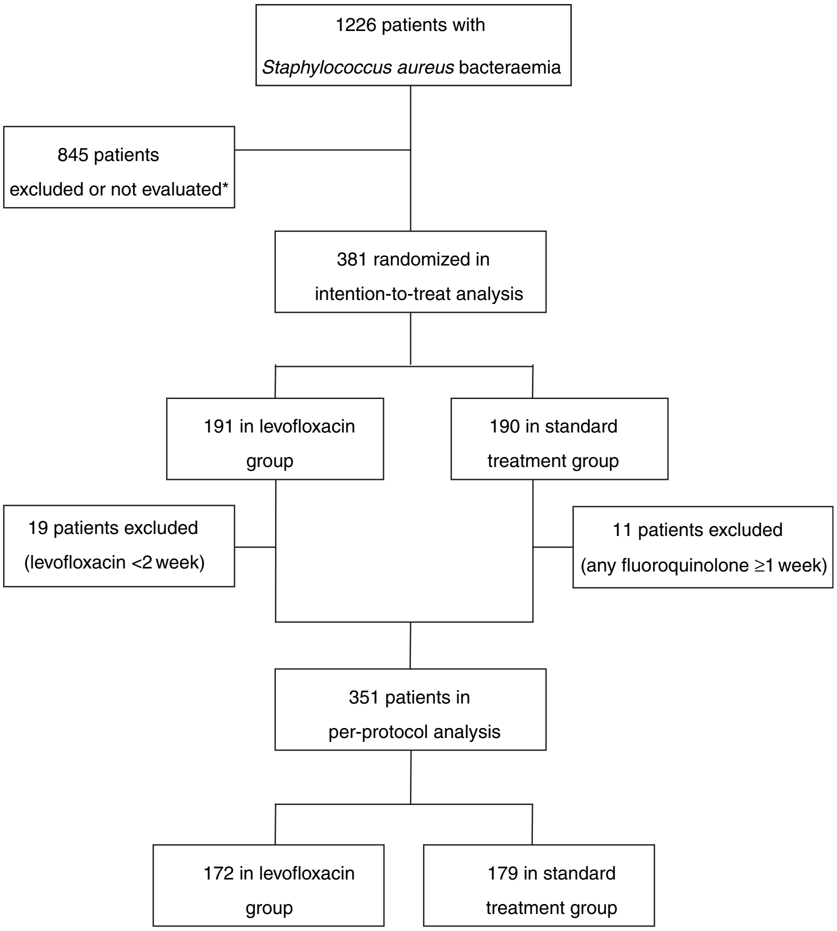

Fig. 1 Study profile. *Not evaluated or excluded patients included 100 patients with failure to supply an informed consent or patient refusal, 48 with neutropenia ($<0.5 \times 10^9$ L^{-1}), 42 deaths prior to randomization, 30 with epilepsy or prior convulsion, 23 with prior fluoroquinolone use for more than 5 days preceding randomization, 15 with a fluoroquinolone resistant strain of *Staphylococcus aureus*, 15 with meningitis, 13 with polymicrobial bacteraemia (≥3 microbes), eight with a positive culture for *S. aureus* only from a central intravenous catheter, seven with another bacteraemia during the previous 28 days, five with bacteraemia due to methicillin-resistant *S. aureus*, three with proven or suspected pregnancy, two prisoners, two breastfeeding, one with a history of allergy to quinolone, one with bacteraemia of borderline oxacillin-resistant *S. aureus*.

Data were analysed from three different patient populations, primarily by intention-to-treat (ITT) analysis with 381 patients. Secondary analysis was performed by per-protocol (PP) (351 patients). Additionally, the length of antibiotic therapy was analysed from a population of which deceased patients were excluded (308 patients). Patients were ineligible for PP analysis if they had received levofloxacin for less than 2 weeks in the levofloxacin group, or any fluoroquinolone for more than 1 week within the first 28 days after randomization in the standard treatment group (Fig. 1).

Statistical analyses were performed with SAS® version 8.2. The primary variable and other categorical variables were analysed with chi-square tests. Odds ratios (OR) with 95% confidence intervals (CI) were calculated to estimate the significance of differences in the two treatment groups. The stratified Cochran–Mantel–Haenszel (CMH) test was used in order to adjust for levofloxacin as confounding factor when effect of rifampicin was analysed. Continuous baseline variables were compared using *t*-test. Decrease in serum CRP concentration was analysed using analysis of variance for repeated measurements (RMANOVA). Mortality and time to defervescence survival estimates were calculated with the Kaplan–Meier method. The log-rank test was used to compare the survival estimates. Survival was calculated from the day of randomization until 3 months. All tests were two-tailed, and a $P < 0.05$ was considered significant. Data were analysed at 4Pharma Ltd (Turku, Finland).

Results

Patient characteristics

During the study period, 1226 patients with SAB were identified (Fig. 1). In total, 381 patients were included in the ITT analysis, with 191 patients in the levofloxacin group and 190 patients in the standard treatment group. All collected data except

Table 1 Characteristics and underlying diseases of 381 patients with *Staphylococcus aureus* bacteraemia

Characteristic	Levofloxacin group (n = 191)	Standard treatment group (n = 190)	Total (n = 381)	OR (95% CI)	P
Age (mean years ± SD)	58 ± 19	58 ± 17	58 ± 19	–	0.97
Male sex	116 (61)	121 (64)	237 (62)	1.13 (0.75–1.72)	0.55
Hospital-acquired	102 (53)	105 (55)	207 (54)	1.08 (0.72–1.61)	0.72
Previous skin disease or wound	94 (49)	108 (57)	202 (53)	1.36 (0.91–2.04)	0.14
Prosthetic or intravascular device	63 (33)	66 (35)	129 (34)	1.08 (0.71–1.65)	0.72
Trauma in previous 2 months	51 (27)	49 (26)	100 (26)	0.95 (0.60–1.51)	0.84
Previous surgery[a]	43 (23)	44 (23)	87 (23)	1.04 (0.64–1.67)	0.88
Central intravenous catheter	30 (16)	22 (12)	52 (14)	0.70 (0.39–1.27)	0.24
Intravenous drug abuse[b]	20 (11)	22 (12)	42 (11)	1.12 (0.59–2.13)	0.73
Corticosteroid use ≥1 month	20 (11)	20 (11)	40 (11)	1.01 (0.52–1.94)	0.99
Alcohol abuse	22 (12)	17 (9)	39 (10)	0.76 (0.39–1.47)	0.41
Immunosuppressive therapy[b]	10 (5)	15 (8)	25 (7)	1.55 (0.68–3.55)	0.30
Cardiovascular disease	84 (44)	93 (49)	177 (47)	1.22 (0.82–1.82)	0.33
Diabetes	50 (26)	53 (28)	103 (27)	1.09 (0.69–1.72)	0.71
Chronic lung disease[c]	30 (16)	40 (21)	70 (18)	1.43 (0.85–2.41)	0.18
Chronic renal failure	29 (15)	30 (16)	59 (16)	1.05 (0.60–1.83)	0.87
Hepatic cirrhosis	28 (15)	28 (15)	56 (15)	1.01 (0.57–1.77)	0.98
Autoimmune disease	24 (13)	20 (11)	44 (12)	0.82 (0.44–1.54)	0.53
Cancer	22 (12)	23 (12)	45 (12)	1.06 (0.57–1.97)	0.86
Haematological malignancy	8 (4)	7 (4)	15 (4)	0.88 (0.31–2.46)	0.80
HIV	5 (3)	3 (2)	8 (2)	0.60 (0.14–2.53)	0.48

Values are expressed as n (%), unless otherwise stated. [a]During 3 months preceding the positive blood culture. [b]During 6 months preceding the positive blood culture. [c]Chronic obstructive pulmonary disease, bronchial asthma and other pulmonary disease.

demographic characteristics were analysed in both ITT and PP populations, but only results from ITT analyses are shown.

Patients in the two groups were well matched with respect to demographic characteristics and predisposing conditions (Table 1). When the underlying diseases were grouped by the predicted prognoses (McCabe's classification) [36], 61% of patients had a nonfatal, 27% had an ultimately fatal and 3% had a rapidly fatal disease. Only 9% of the patients were previously healthy. In both groups the median time from sampling of the first positive blood culture to randomization was 3 days.

Antibiotic treatment

All patients were treated with an antibiotic that was effective against *S. aureus* from the time of the first positive blood culture. In ITT analysis, parenteral cloxacillin or dicloxacillin was given to 150 of 191 patients (79%) in the levofloxacin group and to 135 of 190 patients (71%) in the standard treatment group ($P = 0.09$, OR = 0.67, 95% CI = 0.42–1.07). Only 31 patients (16%) in the levofloxacin group and 42 patients (22%) in the standard treatment group were initially treated with cefuroxime with no significant difference between the groups ($P = 0.15$). The treatment groups differed neither in the use of clindamycin or vancomycin. Rifampicin was given significantly more often to patients in the standard treatment group [146 (77%) of 190 patients] than to patients in levofloxacin group [124 (65%) of 191 patients] ($P = 0.01$, OR = 1.79, 95% CI = 1.14–2.81). Combination therapy with an aminoglycoside was also significantly more common in the standard treatment group [44 (23%) of 190 patients] than in the levofloxacin group [20 (11%) of 191 patients] ($P < 0.001$, OR = 2.58, 95% CI = 1.45–4.57).

The median duration of parenteral antibiotic therapy from randomization was 29 days (interquartile range, 22–36 days) in both groups ($P = 0.76$). Levofloxacin was given for a median of 42 days (interquartile range, 28–58 days). Total duration of antibiotic therapy, including intravenous and oral dosing, was for a median of 72 days (interquartile range, 45–85 days) in the levofloxacin group and 80 days (interquartile range,

42–84 days) in the standard treatment group ($P = 0.90$).

Infection foci

At least one deep infection was detected in 331 patients (87%) during the 3 months follow-up (ITT analysis). Deep infections were definite in 252 patients (76%) and suspected in 79 patients (24%). Most of these (84%) were diagnosed within 1 week of randomization (Table 2). A new deep infection after the first week was found in 33 patients (17%) in the levofloxacin group and in 31 patients (16%) in the standard treatment group ($P = 0.80$). The infection focus was treated with drainage or surgery in 224 patients (59%) with no significant difference between the groups.

Endocarditis was observed in 70 patients (18%) with no significant difference between the study groups (Table 2). Endocarditis was classified as definite in 55 patients (79%) and possible in 15 patients (21%). During the follow-up, five patients (1%) had a new SAB more than 28 days after randomization with no significant difference between the groups. Recurrent SAB was due to a relapse in three patients and reinfection in two patients.

Outcome

No significant differences were observed between the treatment groups in ITT or in PP analyses (Table 3). The case fatality rate at 28 days was 14% in both study arms, and at 3 months 21% in the standard treatment and 18% in the levofloxacin group (ITT analysis) (Table 3, Fig. 2).

In patients with a deep infection, case fatality rate at 3 months was significantly higher amongst those who did not receive rifampicin [25 (38%) of 66 patients] than in patients treated with rifampicin [44 (17%) of 265 patients] ($P < 0.001$, OR = 3.06, 95% CI = 1.69–5.54) (Table 4). However, patients who did not receive rifampicin were significantly older and significantly more often had chronic renal failure, a fatal underlying disease, hospital-acquired SAB, or levofloxacin treatment than did those given rifampicin. In contrast, patients not treated with rifampicin had fewer cases of endocarditis and fewer deep infections per patient.

Mortality in patients who had a deep infection was analysed separately amongst those treated with or without rifampicin (stratified CMH test). This was done as the patients in the standard treatment group were treated with rifampicin significantly more often than the patients in the levofloxacin group ($P = 0.003$) (Table 4). The case fatality rate at 3 months amongst patients with deep infection and rifampicin treatment was 13% (15 of 119 patients) in the levofloxacin group and 20% (29 of 146 patients) in the standard treatment group. Case fatality rates in patients not treated with rifampicin were 37% (16 of 43 patients) and 39% (9 of 23 patients). However, the benefit of levofloxacin was not statistically significant in this stratified analysis either, in which the imbalance in the use of rifampicin was taken into account ($P = 0.16$).

The mean duration of fever (>37.5 °C) was 9 days in both groups (Fig. 2). Decrease rates in serum CRP concentration were similar in both groups (Fig. 2). No significant differences were observed between the treatment groups in the number of patients with leucocytosis, leucopenia, thrombocytopenia, acidosis or liver enzyme elevations (data not shown). There were no significant differences in antibiotic-associated diarrhoea caused by *Clostridium difficile* or allergic reactions between the groups.

Discussion

This is the first clinical trial to evaluate the efficacy of a new fluoroquinolone, with improved Gram-positive activity, combined with standard treatment in SAB. In experimental studies, fluoroquinolone combined with standard therapy has shown improvement in treatment results [26] which could not be confirmed in this clinical trial. New treatment options for bacteraemia caused by MRSA strains would be needed. If fluoroquinolones could be useful in MRSA, bacteraemias cannot be answered by this trial because they were not included. However, resistance to fluoroquinolones has been increasing, especially amongst MRSA strains [38].

Overall, 14% of patients in our trial died within 1 month. This is comparable with the mortality of 17% at 28 days we observed in a nationwide, population-based survey of SAB during 1995–2001 in Finland [39], but clearly lower than the overall mortality of 23–39% generally related to

Table 2 Infection foci of 381 patients with *Staphylococcus aureus* bacteraemia randomized either to standard treatment or combined with levofloxacin

	From randomization to 1 week					From 1 week to 3 months				
Infection focus	Levofloxacin group (n = 191)	Standard treatment group (n = 190)	Total (n = 381)	OR (95% CI)	P	Levofloxacin group (n = 191)	Standard treatment group (n = 190)	Total (n = 381)	OR (95% CI)	P
Skin or soft tissue	113 (59)	131 (69)	244 (64)	1.53 (1.01–2.34)	0.05	7 (4)	3 (2)	10 (3)	0.42 (0.11–1.66)	0.20
Central intravenous catheter	23 (12)	15 (8)	38 (10)	0.63 (0.32–1.24)	0.18	0 (0)	0 (0)	0 (0)	–	–
Deep-seated abscess	76 (40)	75 (40)	151 (40)	0.99 (0.66–1.49)	0.95	4 (2)	14 (7)	18 (5)	3.72 (1.20–11.51)	0.02
Intramuscular	24 (13)	27 (14)	51 (13)	1.15 (0.64–2.08)	0.64	1 (1)	6 (3)	7 (2)	6.20 (0.74–51.96)	0.06
Epidural or CNS	23 (12)	15 (8)	38 (10)	0.63 (0.32–1.24)	0.18	3 (2)	6 (3)	9 (2)	2.04 (0.50–8.29)	0.31
Other[a]	45 (24)	42 (22)	87 (23)	0.92 (0.57–1.49)	0.74	5 (3)	7 (4)	12 (3)	1.42 (0.44–4.57)	0.55
Pneumonia	65 (34)	66 (35)	131 (34)	1.93 (0.68–1.58)	0.89	12 (6)	9 (5)	21 (6)	0.74 (0.31–1.80)	0.51
Osteomyelitis	54 (28)	66 (32)	116 (30)	1.23 (0.79–1.90)	0.36	9 (5)	5 (3)	14 (4)	0.55 (0.18–1.66)	0.28
Prosthetic device[b]	28 (15)	40 (21)	68 (18)	1.55 (0.91–2.64)	0.10	2 (1)	0 (0)	2 (1)	–	–
Endocarditis[c]	30 (16)	37 (20)	67 (18)	1.30 (0.76–2.21)	0.33	1 (1)	2 (1)	3 (1)	–	–
Septic arthritis	18 (9)	28 (15)	46 (12)	1.66 (0.89–3.12)	0.11	2 (1)	2 (1)	4 (1)	–	–
Urinary tract	12 (6)	16 (8)	28 (7)	1.38 (0.63–2.98)	0.42	0 (0)	0 (0)	0 (0)	–	–
Mediastinitis	10 (5)	11 (6)	21 (6)	1.11 (0.46–2.68)	0.81	0 (0)	0 (0)	0 (0)	–	–
Deep infection[d]	155 (81)	166 (87)	321 (84)	1.61 (0.92–2.82)	0.10	33 (17)	31 (16)	64 (17)	0.93 (0.55–1.60)	0.80

Values are expressed as n (%), unless otherwise stated. CNS, central nervous system. [a]Patients with parenchymal, lung, peritoneal, subphrenic, gynaecological and pericardial abscesses, or pleural empyema. [b]Patients with orthopedic, cardiac valve and intravascular devices without peripheral or central intravenous catheters. [c]Possible or definite endocarditis by modified Duke criteria. [d]Patients with deep-seated abscess, pneumonia, osteomyelitis, infection of prosthetic device, endocarditis, septic arthritis, urinary tract infection, mediastinitis, meningitis, septic thrombophlebitis and recurrent *S. aureus* bacteraemia. Each patient has been included once, although some patients had several deep infections at various time-points.

Table 3 Outcome in various subgroups at 28 days and at 3 months for 381 patients with *Staphylococcus aureus* bacteraemia

	At 28 days					At 3 months				
Case fatality rate	Levofloxacin group	Standard treatment group	Total	OR (95% CI)	*P*	Levofloxacin group	Standard treatment group	Total	OR (95% CI)	*P*
Deaths, all	26/191 (14)	27/190 (14)	53/381 (14)	1.05 (0.59–1.88)	0.87	34/191 (18)	39/190 (21)	73/381 (19)	1.19 (0.72–1.99)	0.50
Age										
≤65 years	9/110 (8)	9/113 (8)	18/223 (8)	0.97 (0.37–2.55)	0.95	12/110 (11)	14/113 (12)	26/223 (12)	1.15 (0.51–2.62)	0.73
>65 years	17/81 (21)	18/77 (23)	35/158 (22)	1.15 (0.54–3.67)	0.72	22/81 (27)	25/77 (33)	47/158 (30)	1.29 (0.65–2.55)	0.47
Community-acquired	9/89 (10)	12/85 (14)	21/174 (12)	1.46 (0.58–3.67)	0.42	10/89 (11)	15/85 (18)	25/174 (14)	1.69 (0.71–4.01)	0.23
Hospital-acquired	17/102 (17)	15/105 (14)	32/207 (16)	0.83 (0.39–1.77)	0.64	24/102 (24)	24/105 (23)	48/207 (23)	0.93 (0.50–1.84)	0.91
Diabetes	6/50 (12)	7/53 (13)	13/103 (13)	1.12 (0.35–3.58)	0.85	9/50 (18)	13/53 (25)	22/103 (21)	1.48 (0.57–3.85)	0.42
McCabe's classification										
Healthy or nonfatal	10/134 (8)	12/134 (9)	22/268 (8)	1.22 (0.51–2.93)	0.66	11/134 (8)	18/134 (13)	29/268 (11)	1.74 (0.79–3.83)	0.17
Ultimately or rapidly fatal	16/57 (28)	15/56 (27)	31/113 (27)	0.94 (0.41–2.14)	0.88	23/57 (40)	21/56 (38)	44/113 (39)	0.89 (0.42–1.89)	0.76
Central intravenous catheter	4/23 (17)	1/15 (7)	5/38 (13)	0.34 (0.03–3.38)	0.34	5/23 (22)	2/15 (13)	7/38 (18)	0.55 (0.09–3.31)	0.51
Endocarditis[a]	8/31 (26)	9/39 (23)	17/70 (24)	0.86 (0.29–2.58)	0.79	10/31 (32)	13/39 (33)	23/70 (33)	1.05 (0.38–2.87)	0.92
Deep infection[b]	24/158 (15)	27/168 (16)	51/326 (16)	0.94 (0.51–1.70)	0.83	31/162 (19)	38/169 (23)	69/331 (21)	0.82 (0.48–1.39)	0.45

Values are expressed as *n*/*N* (%), unless otherwise stated. [a]Possible or definite endocarditis by modified Duke criteria. [b]Patients with deep-seated abscess, pneumonia, osteomyelitis, infection of prosthetic device, endocarditis, septic arthritis, urinary tract infection, mediastinitis, meningitis, septic thrombophlebitis and recurrent *S. aureus* bacteraemia.

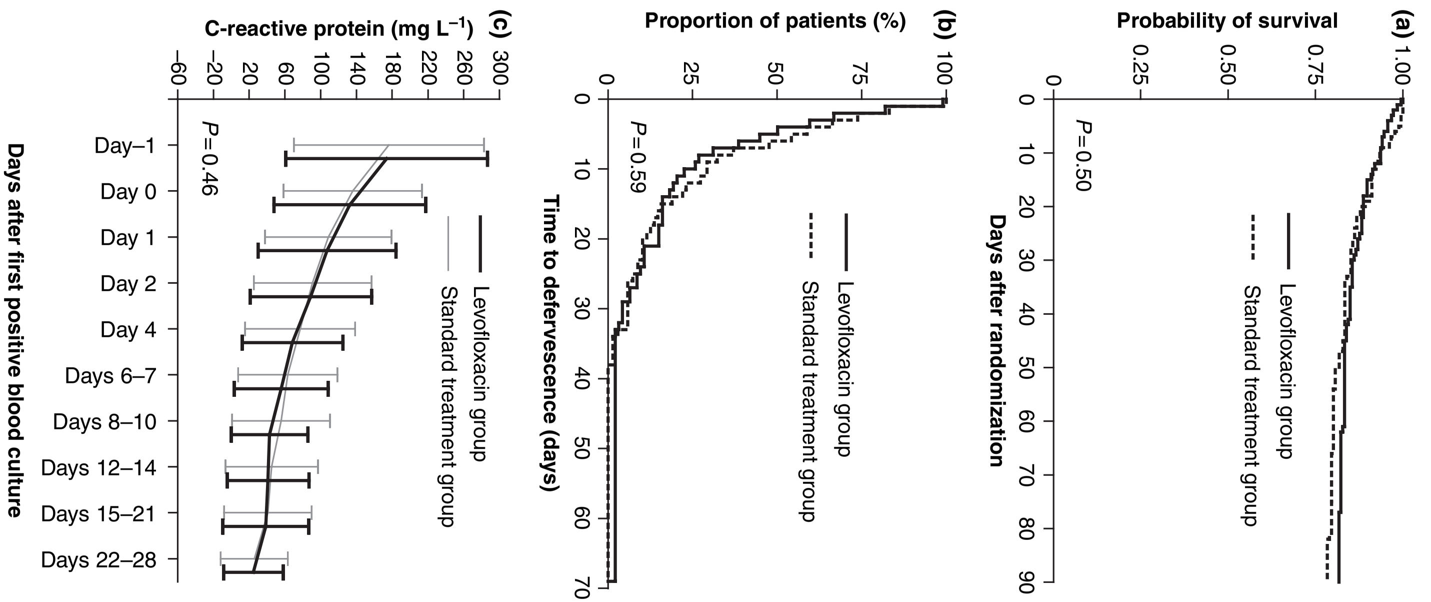

Fig. 2 Kaplan–Meier estimates of the overall survival (panel a) and mean time to defervescence (panel b), and decrease of C-reactive protein concentration (mean ± SD) (panel c) in 381 patients with *Staphylococcus aureus* bacteraemia treated with standard treatment (n = 190) or combined with levofloxacin (n = 191).

Table 4 *Post hoc* analysis of the characteristics and outcome at 3 months of patients with deep infection who received a combination therapy with or without rifampicin

Outcome and variable	Rifampicin (n = 265)	No rifampicin (n = 66)	Total (n = 331)	OR (95% CI)	P
Case fatality rate at 3 months	44 (17)	25 (38)	69 (21)	3.06 (1.69–5.54)	<0.001
Age (mean years ± SD)	57 ± 18	64 ± 19	58 ± 18	–	0.006
Male sex	174 (66)	37 (56)	211 (64)	1.50 (0.87–2.60)	0.15
Hospital-acquired	128 (48)	44 (67)	172 (52)	0.47 (0.27–0.82)	0.008
Diabetes	70 (26)	15 (23)	85 (26)	1.22 (0.65–2.31)	0.54
Chronic renal failure	30 (11)	15 (23)	45 (14)	0.43 (0.22–0.87)	0.02
Hepatic cirrhosis	38 (14)	14 (21)	52 (16)	0.62 (0.31–1.23)	0.17
Cancer	30 (11)	8 (12)	38 (12)	0.93 (0.40–2.13)	0.86
Haematological malignancy	8 (3)	4 (6)	12 (4)	0.48 (0.14–1.65)	0.24
McCabe's classification					
Healthy or nonfatal	199 (75)	36 (55)	235 (71)	1.00 Ref.	–
Ultimately or rapidly fatal	66 (25)	30 (46)	96 (29)	0.40 (0.23–0.70)	<0.001
Central intravenous catheter	16 (6)	6 (9)	22 (7)	0.64 (0.24–1.71)	0.37
Endocarditis[a]	62 (23)	8 (12)	70 (21)	2.21 (1.00–4.89)	0.04
Corticosteroids[b]	106 (40)	21 (32)	127 (38)	1.43 (0.81–2.53)	0.22
Number of deep infections per patient					
≤2	151 (57)	53 (80)	204 (62)	1.00 Ref.	–
>2	114 (43)	13 (20)	127 (38)	3.08 (1.60–5.92)	<0.001
Initial therapy at randomization					
Cloxacillin or dicloxacillin	207 (78)	48 (73)	255 (77)	1.34 (0.72–2.48)	0.35
Cefuroxime	42 (16)	14 (21)	56 (17)	0.70 (0.36–1.38)	0.30
Vancomycin	35 (13)	5 (8)	40 (12)	1.86 (0.70–4.94)	0.21
Levofloxacin	119 (45)	43 (65)	162 (49)	2.29 (1.31–4.02)	0.003

Values are expressed as n (%), unless otherwise stated. [a]Possible or definite endocarditis by modified Duke criteria. [b]During 3 months after randomization.

SAB [1, 6]. Direct comparison of SAB mortality with previous studies is complicated by inconsistent definitions and variable analysis time-points. In some studies only mortality directly attributable to SAB has been calculated [1, 9, 40]. Recent nationwide surveys from low resistance areas in Finland and Denmark [39, 41] suggest that mortality in SAB has decreased during the recent decade, which might be one explanation for the lower mortality in this trial when compared with previous studies.

In the present trial, all patients were followed by an infectious disease specialist, which has been shown to improve the outcome and reduce the number of relapses [6, 42]. However, the low mortality in this trial is in contrast to the high prevalence of deep infections which has earlier been related to higher mortality [6, 9]. The reported frequency of deep infections has varied from 10% to 50%, but it was over 80% in the present trial [9, 13–16]. This difference might be partly explained by different definitions as well as by the high intensity search for deep infections in our trial. Furthermore, in most articles, the incidence of deep infections has not been separately reported or they have been classified into primary and metastatic foci. In another recent study [9], 74% of complications were already present at the time of hospitalization, in accordance with our findings of most deep infections being evident during the first week. These data suggest that infection foci cannot be reliably classed as primary or metastatic and that identification of deep infections might be essential for decreased mortality in SAB.

Intravenous antibiotic treatment is recommended for 4–6 weeks in SAB with a deep infection [17, 43]. In this trial, the duration of parenteral and oral antibiotic therapy was much more prolonged and extended with an average of 77 days. Of all patients, 44% remained on antibiotic treatment at 3 months. This may have contributed to the low (1%) prevalence of SAB recurrences. Significantly higher recurrence rates from 9% to 23%, have been reported in studies with slightly longer follow-up times from 3 to 6 months [5, 7–10, 42, 44].

Data on the effect and recommendations of various antibiotic combinations are controversial,

although they are widely used in complicated SAB. Rifampicin shows excellent antistaphylococcal activity, penetrating well into cells and killing phagocytosed bacteria [22, 23]. The combination of oxacillin and rifampicin has shown a synergistic action *in vitro* when the concentration ratio of oxacillin to rifampicin was low, whereas antagonism occurred with higher ratios [19]. Some small randomized studies have suggested that adding rifampicin to standard treatment improves clinical cure and bacteriological eradication whereas no effect in mortality has been seen [20, 21, 45]. Furthermore, rifampicin is often used in combination with standard treatment in deep-seated abscesses [18], osteomyelitis [45], foreign body infections and endocarditis, or because of poor response to the standard treatment [12, 22–24].

In the current trial, rifampicin was included in the protocol for all patients with deep infection as the ultimate aim was to evaluate whether levofloxacin improved the treatment results when the best therapy was used. Interestingly, if rifampicin was not given, mortality was significantly higher. However, this result must be interpreted with caution, because the trial was not specifically designed to scrutinize the effect of rifampicin. In addition, patients not receiving rifampicin had factors generally associated with higher mortality [6, 14, 40, 44]. These patients were older, more often had hospital-acquired SAB and ultimately or rapidly fatal diseases.

In the levofloxacin group there were significantly more patients with a deep infection not treated with rifampicin (27%) when compared with the standard treatment group (14%). The reasons for not using rifampicin were a concomitant liver disease in 15 cases (nine patients in the levofloxacin group versus six patients in the standard treatment group), a risk of a drug interaction or other decision of the treating doctor in 47 cases (31 patients versus 16 patients), and an early death of the patients in four cases (3 patients versus 1 patient). In patients with deep infection receiving rifampicin, a trend for lower mortality (13%) was observed in the levofloxacin group when compared with the standard treatment group (20%). This difference, however, was not statistically significant. As the present study design did not directly compare levofloxacin to rifampicin, the potential benefit of levofloxacin when rifampicin cannot be used remains to be shown in further prospective studies.

In summary, levofloxacin in combination with standard treatment in SAB did not decrease the mortality or the incidence of deep infections, nor did it speed up recovery. The data indicate that a fluoroquinolone could not be recommended to be combined with the standard treatment of SAB. However, patients with a deep infection appeared to benefit from combination treatment including rifampicin, as suggested also by experimental data.

Conflict of interest statements

Eeva Ruotsalainen has occasionally consulted Aventis Pharma. Asko Järvinen and Ville Valtonen have occasionally consulted several companies, including Aventis Pharma, Bayer, Pfizer, AstraZeneca, Roche, Pharmacia and Merck & Co., Inc. Irma Koivula has occasionally consulted Aventis Pharma and Orion Pharma. Jukka Lumio has occasionally consulted AstraZeneca, Bayer, Pfizer, and Aventis Pharma. Heikki Kauma, Esa Rintala, Pirkko Kotilainen, Martti Vaara, and Jukka Nikoskelainen have no conflicts of interest to declare.

Acknowledgements

This study was supported by an unrestricted grant from Aventis Pharma, and a grant from the special Finnish governmental subsidy for health sciences research. Aventis Pharma also provided levofloxacin for the study patients but had no involvement in the study design, data collection, data interpretation, statistical analysis or writing of this article.

References

1 Conterno LO, Wey SB, Castelo A. Risk factors for mortality in *Staphylococcus aureus* bacteremia. *Infect Control Hosp Epidemiol* 1998; **19:** 32–7.

2 Julander I, Svanbom M. Prediction of staphylococcal etiology among patients with septicemia with or without endocarditis by multivariate statistical methods. *Scand J Infect Dis* 1985; **17:** 37–46.

3 Kuikka A, Valtonen VV. Improved outcome of *Staphylococcus aureus* bacteremia. *Infect Dis Clin Pract* 1994; **3:** 282–7.

4 Watanakunakorn C, Chan SJ, Demarco DG, Palmer JA. *Staphylococcus aureus* bacteremia: significance of hyperbilirubinemia. *Scand J Infect Dis* 1987; **19:** 195–203.

5 Soriano A, Martinez JA, Mensa J *et al.* Pathogenic significance of methicillin resistance for patients with *Staphylococcus aureus* bacteremia. *Clin Infect Dis* 2000; **30:** 368–73.

6 Mylotte JM, Tayara A. *Staphylococcus aureus* bacteremia: predictors of 30-day mortality in a large cohort. *Clin Infect Dis* 2000; **31:** 1170–4.

7 Johnson LB, Almoujahed MO, Ilg K, Maolood L, Khatib R. *Staphylococcus aureus* bacteremia: compliance with standard treatment, long-term outcome and predictors of relapse. *Scand J Infect Dis* 2003; **35:** 782–9.

8 Jensen AG, Wachmann CH, Espersen F, Scheibel J, Skinhoj P, Frimodt-Moller N. Treatment and outcome of *Staphylococcus aureus* bacteremia: a prospective study of 278 cases. *Arch Intern Med* 2002; **162:** 25–32.

9 Fowler VG Jr, Olsen MK, Corey GR *et al.* Clinical identifiers of complicated *Staphylococcus aureus* bacteremia. *Arch Intern Med* 2003; **163:** 2066–72.

10 Chang FY, Peacock JE Jr , Musher DM *et al. Staphylococcus aureus* bacteremia: recurrence and the impact of antibiotic treatment in a prospective multicenter study. *Medicine (Baltimore)* 2003; **82:** 333–9.

11 Jensen AG. Importance of focus identification in the treatment of *Staphylococcus aureus* bacteraemia. *J Hosp Infect* 2002; **52:** 29–36.

12 Lowy FD. *Staphylococcus aureus* infections. *N Engl J Med* 1998; **339:** 520–32.

13 Mylotte JM, McDermott C, Spooner JA. Prospective study of 114 consecutive episodes of *Staphylococcus aureus* bacteremia. *Rev Infect Dis* 1987; **9:** 891–907.

14 Ringberg H, Thoren A, Lilja B. Metastatic complications of *Staphylococcus aureus* septicemia. To seek is to find. *Infection* 2000; **28:** 132–6.

15 Lautenschlager S, Herzog C, Zimmerli W. Course and outcome of bacteremia due to *Staphylococcus aureus*: evaluation of different clinical case definitions. *Clin Infect Dis* 1993; **16:** 567–73.

16 Fatkenheuer G, Preuss M, Salzberger B *et al.* Long-term outcome and quality of care of patients with *Staphylococcus aureus* bacteremia. *Eur J Clin Microbiol Infect Dis* 2004; **23:** 157–62.

17 Petti CA, Fowler VG Jr. *Staphylococcus aureus* bacteremia and endocarditis. *Infect Dis Clin North Am* 2002; **16:** 413–35.

18 Tan JS, File TM Jr. Management of staphylococcal and streptococcal infections. *Clin Podiatr Med Surg* 1996; **13:** 793–816.

19 Van der Auwera P, Klastersky J. In vitro study of the combination of rifampicin with oxacillin against *Staphylococcus aureus*. *Rev Infect Dis* 1983; **5** (Suppl.): S509–14.

20 Van der Auwera P, Meunier-Carpentier F, Klastersky J. Clinical study of combination therapy with oxacillin and rifampicin for staphylococcal infections. *Rev Infect Dis* 1983; **5** (Suppl.): S515–22.

21 Van der Auwera P, Klastersky J, Thys JP, Meunier-Carpentier F, Legrand JC. Double-blind, placebo-controlled study of oxacillin combined with rifampicin in the treatment of staphylococcal infections. *Antimicrob Agents Chemother* 1985; **28:** 467–72.

22 Craig WA. Rifampicin and related drugs. In: Gorbach SL, Bartlett JG, Blacklow NR, eds. *Infectious Diseases*. Philadelphia, PA: Lippincott Williams & Wilkins, 2004; 277–80.

23 Calfee DP. Rifamycins. In: Mandell GL, Bennett JE, Dolin R, eds. *Mandell, Douglas, and Bennett's Principles and Practice of Infectious Diseases*. Philadelphia, PA: Churchill Livingstone, 2005; 374–87.

24 Kapusnik JE, Parenti F, Sande MA. The use of rifampicin in staphylococcal infections – a review. *J Antimicrob Chemother* 1984; **13** (Suppl.): 61–6.

25 Fu KP, Lafredo SC, Foleno B *et al.* In vitro and in vivo antibacterial activities of levofloxacin (l-ofloxacin), an optically active ofloxacin. *Antimicrob Agents Chemother* 1992; **36:** 860–6.

26 Rohner P, Herter C, Auckenthaler R, Pechere JC, Waldvogel FA, Lew DP. Synergistic effect of quinolones and oxacillin on methicillin-resistant *Staphylococcus* species. *Antimicrob Agents Chemother* 1989; **33:** 2037–41.

27 Dworkin RJ, Lee BL, Sande MA, Chambers HF. Treatment of right-sided *Staphylococcus aureus* endocarditis in intravenous drug users with ciprofloxacin and rifampicin. *Lancet* 1989; **2:** 1071–3.

28 Heldman AW, Hartert TV, Ray SC *et al.* Oral antibiotic treatment of right-sided staphylococcal endocarditis in injection drug users: prospective randomized comparison with parenteral therapy. *Am J Med* 1996; **101:** 68–76.

29 Zimmerli W, Widmer AF, Blatter M, Frei R, Ochsner PE. Role of rifampicin for treatment of orthopedic implant-related staphylococcal infections: a randomized controlled trial. Foreign-Body Infection (FBI) Study Group. *JAMA* 1998; **279:** 1537–41.

30 Drancourt M, Stein A, Argenson JN, Roiron R, Groulier P, Raoult D. Oral treatment of *Staphylococcus* spp. infected orthopaedic implants with fusidic acid or ofloxacin in combination with rifampicin. *J Antimicrob Chemother* 1997; **39:** 235–40.

31 Schrenzel J, Harbarth S, Schockmel G *et al.* A randomized clinical trial to compare fleroxacin-rifampicin with flucloxacillin or vancomycin for the treatment of staphylococcal infection. *Clin Infect Dis* 2004; **39:** 1285–92.

32 Libman H, Arbeit RD. Complications associated with *Staphylococcus aureus* bacteremia. *Arch Intern Med* 1984; **144:** 541–5.

33 Mermel LA, Farr BM, Sherertz RJ *et al.* Guidelines for the management of intravascular catheter-related infections. *Clin Infect Dis* 2001; **32:** 1249–72.

34 Rahal JJ Jr, Chan YK, Johnson G. Relationship of staphylococcal tolerance, teichoic acid antibody, and serum bactericidal activity to therapeutic outcome in *Staphylococcus aureus* bacteremia. *Am J Med* 1986; **81:** 43–52.

35 Moreillon P, Que YA. Infective endocarditis. *Lancet* 2004; **363:** 139–49.

36 McCabe WR, Jackson GG. Gram negative bacteraemia 1. Etiology and ecology. *Arch Intern Med* 1962; **110:** 847–55.

37 Li JS, Sexton DJ, Mick N *et al.* Proposed modifications to the Duke criteria for the diagnosis of infective endocarditis. *Clin Infect Dis* 2000; **30:** 633–8.

38 Naimi TS, LeDell KH, Como-Sabetti K *et al.* Comparison of community- and health care-associated methicillin-resistant *Staphylococcus aureus* infection. *JAMA* 2003; **290:** 2976–84.

39 Lyytikäinen O, Ruotsalainen E, Järvinen A, Valtonen V, Ruutu P. Trends and outcome of nosocomial and community-acquired bloodstream infections due to *Staphylococcus aureus* in Finland, 1995–2001. *Eur J Clin Microbiol Infect Dis* 2005; **24:** 399–404.

40 Kim SH, Park WB, Lee KD *et al.* Outcome of *Staphylococcus aureus* bacteremia in patients with eradicable foci versus noneradicable foci. *Clin Infect Dis* 2003; **37:** 794–9.

41 Benfield T, Espersen F, Frimodt-Moller N *et al.* Trends in incidence and mortality among 23962 adult cases of *Staphylococcus aureus* bacteremia from 1971 through 2000. In: *Program and Abstracts of the 44th Interscience Conference on Antimicrobial Agents and Chemotherapy*, Washington, DC, October 30–November 2, 2004: L-357 (abstract).

42 Fowler VG Jr, Sanders LL, Sexton DJ *et al.* Outcome of *Staphylococcus aureus* bacteremia according to compliance with recommendations of infectious diseases specialists: experience with 244 patients. *Clin Infect Dis* 1998; **27:** 478–86.

43 Moreillon P, Que YA, Glauser MP. *Staphylococcus aureus* (including staphylococcal toxic shock). In: Mandell GL, Bennett JE, Dolin R, eds. *Mandell, Douglas, and Bennett's Principles and Practice of Infectious Diseases*. Philadelphia, PA: Churchill Livingstone, 2005; 2321–51.

44 Chang FY, MacDonald BB, Peacock JE Jr *et al.* A prospective multicenter study of *Staphylococcus aureus* bacteremia: incidence of endocarditis, risk factors for mortality, and clinical impact of methicillin resistance. *Medicine (Baltimore)* 2003; **82:** 322–32.

45 Norden CW, Bryant R, Palmer D, Montgomerie JZ, Wheat J. Chronic osteomyelitis caused by *Staphylococcus aureus*: controlled clinical trial of nafcillin therapy and nafcillin-rifampicin therapy. *South Med J* 1986; **79:** 947–51.

Correspondence: Eeva Ruotsalainen, MD, Division of Infectious Diseases, Department of Medicine, Helsinki University Central Hospital, PO Box 348 00029 HUS, Helsinki, Finland.
(fax: +358 9 471 75945; e-mail: eeva.ruotsalainen@hus.fi).
